# *catena*-Poly[[trimethyl­tin(IV)]-μ-cyclo­hex-3-ene-1-carboxyl­ato]

**DOI:** 10.1107/S1600536809054403

**Published:** 2009-12-24

**Authors:** Yun Ren, Rufen Zhang, Yang Shi

**Affiliations:** a College of Chemistry and Chemical Engineering, Liaocheng University, Shandong 252059, People’s Republic of China; b Department of Chemistry, Liaocheng University, Liaocheng 252059, People’s Republic of China

## Abstract

The title compound, [Sn(CH_3_)_3_(C_7_H_9_O_2_)]_*n*_, forms an extended zigzag chain structure propagating parallel to [010]. The Sn atom is in a slightly distorted trigonal-bipyramidal coordination environment with two carboxyl­ate O atoms in the axial and the three methyl groups in equatorial sites. The cyclo­hexene ring has a distorted half-boat conformation. There is an intra­molecular C—H⋯O hydrogen bond.

## Related literature

For related structures, see: Murugavel *et al.* (2001 ▶); Ma *et al.* (2006 ▶). For puckering parameters, see: Cremer & Pople (1975 ▶).
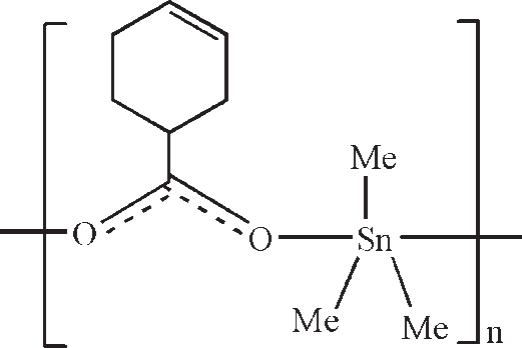


## Experimental

### 

#### Crystal data

[Sn(CH_3_)_3_(C_7_H_9_O_2_)]
*M* *_r_* = 288.93
Monoclinic, 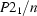

*a* = 11.3022 (15) Å
*b* = 9.8469 (14) Å
*c* = 12.1468 (18) Å
β = 112.148 (2)°
*V* = 1252.1 (3) Å^3^
*Z* = 4
Mo *K*α radiation
μ = 2.01 mm^−1^
*T* = 298 K
0.45 × 0.36 × 0.33 mm

#### Data collection

Siemens SMART CCD area-detector diffractometer
Absorption correction: multi-scan (*SADABS*; Sheldrick, 1996 ▶) *T*_min_ = 0.464, *T*_max_ = 0.556
6095 measured reflections
2193 independent reflections
1669 reflections with *I* > 2σ(*I*)
*R*_int_ = 0.033

#### Refinement

*R*[*F* ^2^ > 2σ(*F* ^2^)] = 0.035
*wR*(*F* ^2^) = 0.101
*S* = 1.08
2193 reflections
118 parameters
H-atom parameters constrained
Δρ_max_ = 1.53 e Å^−3^
Δρ_min_ = −0.49 e Å^−3^

### 

Data collection: *SMART* (Siemens, 1996 ▶); cell refinement: *SAINT* (Siemens, 1996 ▶); data reduction: *SAINT*; program(s) used to solve structure: *SHELXS97* (Sheldrick, 2008 ▶); program(s) used to refine structure: *SHELXL97* (Sheldrick, 2008 ▶); molecular graphics: *SHELXTL* (Sheldrick, 2008 ▶); software used to prepare material for publication: *SHELXTL*.

## Supplementary Material

Crystal structure: contains datablocks I, global. DOI: 10.1107/S1600536809054403/bx2254sup1.cif

Structure factors: contains datablocks I. DOI: 10.1107/S1600536809054403/bx2254Isup2.hkl

Additional supplementary materials:  crystallographic information; 3D view; checkCIF report

## Figures and Tables

**Table 1:** Hydrogen-bond geometry (Å, °)

*D*—H⋯*A*	*D*—H	H⋯*A*	*D*⋯*A*	*D*—H⋯*A*
C3—H3*B*⋯O2	0.97	2.60	2.926 (10)	100

**Fig. 1.**
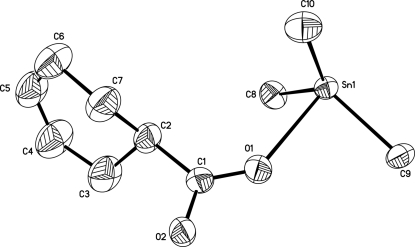
Part of the polymericstructure and the atom-numbering scheme of the title compound. Displacementellipsoids are shown at the 30% probability level and H atoms have been omitted for clarity.

**Fig. 2.**
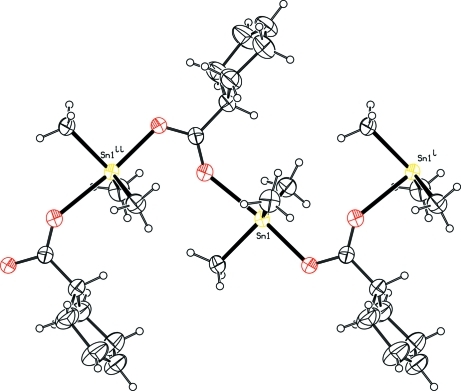
Part of the structure of (I) showing the one-dimensional chain along the b axis[symmetry codes: (i) 1/2-x,-1/2+y, 1/2-z; (ii) 1/2-x,1/2+y,1/2-z].

## supplementary crystallographic information

### Comment

Organotin complexes are attracting more and more attention because of their considerable structural diversity and interesting topologies (Murugavel *et al.* 2001). Herein, we report the crystal structure of the title compound. The title compound, which is shown in Fig.1 forms an infinite zigzag one-dimensional polymeric chain structure.The Sn atom is in a slightly distorted trigonal-bipyramidal coordination environment with two carboxylate O atoms in the axial sites and three methyl groups in equatorial site.The Sn—O, Sn—C bond lenghts and the O—Sn···O bond angles are close to the reported organotin compounds (Ma *et al.* 2006). The cyclohexene ring is in a distorted half-boat conformation, the ring-puckering parameters (Cremer & Pople, 1975) are q_2_ = 0.380 (1) Å , q_3_ = -0.314 (1) Å , Q = 0.492 (9) ° and φ_2_ = 165.1 (2)°. There is an intramolecular C—H···O hydrogen bond (H3B—O2 2.60 , C3—O2 2.926 (10) Å, C3—H3B···O2 100°).

### Experimental

The reaction was carried out under nitrogen atmosphere. 3-cyclohexene-1-carboxylic acid (1 mmol) and sodium ethoxide (1 mmol) were added to a stirred solution of benzene (30 ml) in a Schlenk flask and stirred for 0.5 h. trimethyltin chloride (1 mmol) was then added to the reactor and the reaction mixture was stirred for 12 h at room temperature. The resulting clear solution was evaporated under vacuum. The product was crystallized from ether to yield colorless blocks of the title compound (yield 86% m.p.448 K). Anal. Calcd (%) for C_10_H_18_O_2_Sn_1_ (Mr = 288.93): C, 41.57; H, 6.28; Found (%): C, 41.76; H, 6.01.

### Refinement

All H atoms were positioned geometrically and constrained to ride on their parent atoms, with C—H = 0.93–0.98 Å, and with *U*_iso_(H) = 1.5*U*_eq_(C) for methyl H and 1.2*U*_eq_(C), for the others.

### Crystal data

**Table:**

[Sn(CH_3_)_3_(C_7_H_9_O_2_)]	*F*(000) = 576
*M**_r_* = 288.93	*D*_x_ = 1.533 Mg m^−^^3^
Monoclinic, *P*2_1_/*n*	Mo *K*α radiation, λ = 0.71073 Å
Hall symbol: -P 2yn	Cell parameters from 2901 reflections
*a* = 11.3022 (15) Å	θ = 2.8–25.7°
*b* = 9.8469 (14) Å	µ = 2.01 mm^−^^1^
*c* = 12.1468 (18) Å	*T* = 298 K
β = 112.148 (2)°	Block, colourless
*V* = 1252.1 (3) Å^3^	0.45 × 0.36 × 0.33 mm
*Z* = 4	

### Data collection

**Table:**

Siemens SMART CCD area-detector diffractometer	2193 independent reflections
Radiation source: fine-focus sealed tube	1669 reflections with *I* > 2σ(*I*)
graphite	*R*_int_ = 0.033
φ and ω scans	θ_max_ = 25.0°, θ_min_ = 2.1°
Absorption correction: multi-scan (*SADABS*; Sheldrick, 1996)	*h* = −13→13
*T*_min_ = 0.464, *T*_max_ = 0.556	*k* = −10→11
6095 measured reflections	*l* = −14→9

### Refinement

**Table:**

Refinement on *F*^2^	Primary atom site location: structure-invariant direct methods
Least-squares matrix: full	Secondary atom site location: difference Fourier map
*R*[*F*^2^ > 2σ(*F*^2^)] = 0.035	Hydrogen site location: inferred from neighbouring sites
*wR*(*F*^2^) = 0.101	H-atom parameters constrained
*S* = 1.08	*w* = 1/[σ^2^(*F*_o_^2^) + (0.0436*P*)^2^ + 2.3141*P*] where *P* = (*F*_o_^2^ + 2*F*_c_^2^)/3
2193 reflections	(Δ/σ)_max_ = 0.001
118 parameters	Δρ_max_ = 1.53 e Å^−^^3^
0 restraints	Δρ_min_ = −0.49 e Å^−^^3^

### Fractional atomic coordinates and isotropic or equivalent isotropic displacement parameters (Å^2^)

**Table:**

	*x*	*y*	*z*	*U*_iso_*/*U*_eq_	
Sn1	0.17568 (3)	0.09824 (4)	0.23917 (3)	0.04584 (17)	
O1	0.3014 (4)	0.2882 (4)	0.2205 (5)	0.0699 (12)	
O2	0.4496 (4)	0.4411 (4)	0.2415 (4)	0.0652 (12)	
C1	0.4091 (6)	0.3183 (6)	0.2231 (6)	0.0574 (15)	
C2	0.4961 (6)	0.2138 (7)	0.2052 (7)	0.0694 (18)	
H2	0.4529	0.1255	0.1915	0.083*	
C3	0.5310 (8)	0.2458 (9)	0.1026 (8)	0.101 (3)	
H3A	0.4539	0.2451	0.0313	0.121*	
H3B	0.5654	0.3373	0.1125	0.121*	
C4	0.6246 (10)	0.1532 (11)	0.0847 (10)	0.118 (3)	
H4	0.6277	0.1437	0.0097	0.142*	
C5	0.7028 (9)	0.0848 (9)	0.1741 (10)	0.098 (3)	
H5	0.7612	0.0275	0.1603	0.118*	
C6	0.7047 (9)	0.0922 (9)	0.2906 (10)	0.104 (3)	
H6A	0.7920	0.1077	0.3449	0.125*	
H6B	0.6781	0.0051	0.3106	0.125*	
C7	0.6205 (7)	0.2020 (9)	0.3105 (8)	0.091 (2)	
H7A	0.6032	0.1806	0.3809	0.109*	
H7B	0.6650	0.2883	0.3236	0.109*	
C8	0.1852 (7)	0.0177 (7)	0.0817 (6)	0.0684 (18)	
H8A	0.2669	−0.0244	0.0994	0.103*	
H8B	0.1742	0.0896	0.0253	0.103*	
H8C	0.1189	−0.0486	0.0489	0.103*	
C9	0.0327 (6)	0.2468 (6)	0.2198 (7)	0.072 (2)	
H9A	0.0032	0.2384	0.2840	0.109*	
H9B	−0.0375	0.2333	0.1456	0.109*	
H9C	0.0679	0.3358	0.2212	0.109*	
C10	0.3183 (7)	0.0735 (7)	0.4101 (6)	0.075 (2)	
H10A	0.3965	0.0455	0.4031	0.113*	
H10B	0.2917	0.0057	0.4528	0.113*	
H10C	0.3315	0.1581	0.4523	0.113*	

### Atomic displacement parameters (Å^2^)

**Table:**

	*U*^11^	*U*^22^	*U*^33^	*U*^12^	*U*^13^	*U*^23^
Sn1	0.0474 (3)	0.0394 (2)	0.0517 (3)	0.00229 (17)	0.01983 (19)	0.00063 (18)
O1	0.065 (3)	0.049 (2)	0.105 (4)	−0.003 (2)	0.043 (3)	−0.001 (3)
O2	0.063 (3)	0.046 (2)	0.102 (4)	−0.0029 (19)	0.048 (3)	−0.007 (2)
C1	0.063 (4)	0.046 (3)	0.070 (4)	0.004 (3)	0.032 (3)	0.003 (3)
C2	0.066 (4)	0.053 (4)	0.098 (5)	0.005 (3)	0.041 (4)	−0.009 (4)
C3	0.114 (6)	0.101 (6)	0.110 (7)	0.027 (5)	0.068 (6)	−0.007 (5)
C4	0.129 (8)	0.132 (8)	0.110 (8)	0.048 (7)	0.065 (7)	−0.001 (7)
C5	0.090 (6)	0.092 (6)	0.123 (8)	0.026 (5)	0.054 (6)	−0.011 (6)
C6	0.097 (6)	0.092 (6)	0.121 (8)	0.033 (5)	0.039 (6)	0.008 (6)
C7	0.085 (5)	0.087 (6)	0.103 (6)	0.021 (5)	0.040 (5)	0.005 (5)
C8	0.077 (4)	0.073 (4)	0.059 (4)	−0.006 (4)	0.030 (4)	−0.006 (4)
C9	0.061 (4)	0.049 (3)	0.111 (6)	0.012 (3)	0.036 (4)	0.016 (4)
C10	0.079 (5)	0.080 (5)	0.054 (4)	0.002 (4)	0.009 (4)	0.006 (4)

### Geometric parameters (Å, °)

**Table:**

Sn1—C10	2.108 (7)	C5—C6	1.408 (14)
Sn1—C8	2.110 (6)	C5—H5	0.9300
Sn1—C9	2.126 (6)	C6—C7	1.519 (11)
Sn1—O2^i^	2.169 (4)	C6—H6A	0.9700
Sn1—O1	2.411 (4)	C6—H6B	0.9700
O1—C1	1.241 (7)	C7—H7A	0.9700
O2—C1	1.282 (7)	C7—H7B	0.9700
O2—Sn1^ii^	2.169 (4)	C8—H8A	0.9600
C1—C2	1.495 (8)	C8—H8B	0.9600
C2—C3	1.475 (10)	C8—H8C	0.9600
C2—C7	1.506 (10)	C9—H9A	0.9600
C2—H2	0.9800	C9—H9B	0.9600
C3—C4	1.474 (11)	C9—H9C	0.9600
C3—H3A	0.9700	C10—H10A	0.9600
C3—H3B	0.9700	C10—H10B	0.9600
C4—C5	1.300 (13)	C10—H10C	0.9600
C4—H4	0.9300		
			
C10—Sn1—C8	124.6 (3)	C6—C5—H5	118.0
C10—Sn1—C9	117.1 (3)	C5—C6—C7	115.0 (8)
C8—Sn1—C9	117.0 (3)	C5—C6—H6A	108.5
C10—Sn1—O2^i^	95.7 (2)	C7—C6—H6A	108.5
C8—Sn1—O2^i^	95.1 (2)	C5—C6—H6B	108.5
C9—Sn1—O2^i^	90.2 (2)	C7—C6—H6B	108.5
C10—Sn1—O1	85.6 (2)	H6A—C6—H6B	107.5
C8—Sn1—O1	88.4 (2)	C2—C7—C6	111.1 (7)
C9—Sn1—O1	84.6 (2)	C2—C7—H7A	109.4
O2^i^—Sn1—O1	174.62 (14)	C6—C7—H7A	109.4
C1—O1—Sn1	142.2 (4)	C2—C7—H7B	109.4
C1—O2—Sn1^ii^	119.1 (4)	C6—C7—H7B	109.4
O1—C1—O2	120.8 (5)	H7A—C7—H7B	108.0
O1—C1—C2	121.6 (6)	Sn1—C8—H8A	109.5
O2—C1—C2	117.6 (5)	Sn1—C8—H8B	109.5
C3—C2—C1	112.1 (6)	H8A—C8—H8B	109.5
C3—C2—C7	105.8 (6)	Sn1—C8—H8C	109.5
C1—C2—C7	112.6 (6)	H8A—C8—H8C	109.5
C3—C2—H2	108.8	H8B—C8—H8C	109.5
C1—C2—H2	108.8	Sn1—C9—H9A	109.5
C7—C2—H2	108.8	Sn1—C9—H9B	109.5
C4—C3—C2	115.3 (8)	H9A—C9—H9B	109.5
C4—C3—H3A	108.4	Sn1—C9—H9C	109.5
C2—C3—H3A	108.4	H9A—C9—H9C	109.5
C4—C3—H3B	108.4	H9B—C9—H9C	109.5
C2—C3—H3B	108.4	Sn1—C10—H10A	109.5
H3A—C3—H3B	107.5	Sn1—C10—H10B	109.5
C5—C4—C3	119.8 (9)	H10A—C10—H10B	109.5
C5—C4—H4	120.1	Sn1—C10—H10C	109.5
C3—C4—H4	120.1	H10A—C10—H10C	109.5
C4—C5—C6	124.1 (8)	H10B—C10—H10C	109.5
C4—C5—H5	118.0		

Symmetry codes: (i) −*x*+1/2, *y*−1/2, −*z*+1/2; (ii) −*x*+1/2, *y*+1/2, −*z*+1/2.

### Hydrogen-bond geometry (Å, °)

**Table:**

*D*—H···*A*	*D*—H	H···*A*	*D*···*A*	*D*—H···*A*
C3—H3B···O2	0.97	2.60	2.926 (10)	100

## References

[bb1] Cremer, D. & Pople, J. A. (1975). *J. Am. Chem. Soc.* **97**, 1354–1358.

[bb2] Ma, C., Li, J., Zhang, R. & Wang, D. (2006). *J. Organomet. Chem.* **691**, 1713–1721.

[bb3] Murugavel, R., Baheti, K. & Anantharaman, G. (2001). *Inorg. Chem.* **40**, 6870–6878. 10.1021/ic010519b 11754266

[bb4] Sheldrick, G. M. (1996). *SADABS* University of Göttingen, Germany.

[bb5] Sheldrick, G. M. (2008). *Acta Cryst.* A**64**, 112–122. 10.1107/S0108767307043930 18156677

[bb6] Siemens (1996). *SMART* and *SAINT* Siemens Analytical X-ray Instruments Inc., Madison, Wisconsin, USA.

